# Bio-Functional Activities of Tuscan Bee Pollen

**DOI:** 10.3390/antiox12010115

**Published:** 2023-01-03

**Authors:** Elisa Chelucci, Carolina Chiellini, Andrea Cavallero, Morena Gabriele

**Affiliations:** Institute of Agricultural Biology and Biotechnology, Italian National Research Council, Via Moruzzi 1, 56124 Pisa, Italy

**Keywords:** bee pollen, bioactive compounds, yeast, antioxidant activity, hemolysis test, CAA-RBC, human colonic adenocarcinoma cells (HT-29), inflammation

## Abstract

Bee pollen represents one of the most complete natural foods playing an important role in the diet for its health qualities and therapeutic properties. This work aimed to characterize a Tuscan bee pollen by evaluating its phytochemical profile and the in vitro and ex vivo antioxidant activities. The isolation and taxonomic and functional characterization of yeasts in the sample has been also conducted. Finally, the pollen anti-inflammatory potential has been assessed on a TNFα-inflamed human colorectal adenocarcinoma cell line (HT-29). Our results highlighted a good phytochemical composition in terms of polyphenols, flavonoids, flavonols, monomeric anthocyanins, and carotenoids. In addition, we detected good antioxidant activity and radical scavenging capacity by in vitro and ex vivo assays, as well as good antioxidant activity by isolated yeasts. Data showed no cytotoxic effects of bee pollen extracts, with average viability values >80% at each tested dose. Moreover, TNFα treatment did not affect HT-29 viability while upregulating IL-8, COX-2, and ICAM-1 gene expression, otherwise reduced by both doses of bee pollen. In conclusion, our sample represents an interesting functional food and a potential probiotic product, having high phytochemical compound levels and good antioxidant activities, as well as anti-inflammatory effects on the TNFα-inflamed HT-29 cell line.

## 1. Introduction

For centuries, apicultural products have been used in phytotherapy and alternative medicine and played an important role in the human diet for their health qualities and nutritional implications [1,2,3,4,5]. Among apicultural products, bee pollen represents one of the most complete natural foods, and a great source of energy for human nutrition, being rich in nutrients and bioactive compounds [1,2,6]. 

Bee pollen consists of small colored pollen grains harvested by honeybees (*Apis mellifera*) from flowering plants anthers using beehives equipped with bottom-fitted pollen traps so that honeybees lose pollen loads before entering [2,7,8,9]. Among bee pollen types, the monofloral pollen consists of a minimum of 45% pollen grains from one specific plant species, while polyfloral one is composed of pollen grains from various plant species [10]. Pollen grains have different colors, sizes, weights, and shapes depending on the botanical and geographical origins of plants [1,2,11,12]. Bee pollen might contain nectar, salivary secretions, and substances produced by honeybees, which package it with enzymatic processes in pollen loads before coming back to the hive [2,3,6,11], where they store it separately from the nectar cells [7].

Currently, bee pollen is considered a functional food owing to its high nutritional value and physiological properties [2,3,8,13]. A great number of different substances are contained in bee pollen grains, representing a good diet supplement and providing high levels of macro and micronutrients such as carbohydrates (13–55%), proteins (10–40%), including free amino acids enzymes and cofactors, lipids (1–13%), vitamins (especially A, B, C, and E), and minerals [1,2,3,8,9,11,12]. Additionally, bee pollen represents a good source of bioactive compounds, particularly polyphenols and carotenoids, exerting excellent health promoting effects [1]. 

Several studies have pointed out the positive therapeutic effects of bee pollen, such as its antioxidant, anti-inflammatory, anti-mutagenic, anti-allergic, antimicrobial, and antitumor properties [1,2,3,8,11,13]. Most of these bioactivities have been linked to micronutrients, essential amino acids, antioxidant enzymes, and phenolic compounds. Moreover, the presence of a complex microbial community, consisting of both bacteria and yeasts, has been the focus of many recent studies aimed at discovering the beneficial role of a such component in bee pollen [14].

This work aimed to characterize a Tuscan bee pollen, harvested from Bagni di Lucca (LU, Italy), by evaluating its phytochemical profile, in vitro and ex vivo antioxidant activities, as well as the yeast community inhabiting it. Finally, we also assessed its anti-inflammatory potential on an inflamed human colorectal adenocarcinoma cell line (HT-29) exposed to the tumor necrosis factor-alpha (TNF-α) used to induce inflammation. 

## 2. Materials and Methods

### 2.1. Chemicals and Reagents

All standards and reagents were of analytical grade. Folin-Ciocalteu reagent, saline solution (NaCl 0.9% *w/v*), sodium carbonate, ethylenediaminetetraacetic acid (EDTA), sodium acetate, potassium chloride, gallic acid, sodium hydroxide, quercetin dihydrate, catechin hydrate, phosphate buffer saline (PBS) tablets, 6-hydroxy-2,5,7,8-tetramethylchromane-2-carboxylic acid (Trolox), potassium persulfate, 2,2′-azinobis(3-ethylbenzthiazoline-6-sulphonic acid) diammonium salt (ABTS), 2,2-diphenyl-1-picrylhydrazyl (DPPH), 2,4,6-Tri(2-pyridyl)-s-triazine (TPTZ), ferric chloride hexahydrate (FeCl_3_·6H_2_O), ferrous sulfate heptahydrate (FeSO_4_·7H_2_O), ferrozine, sodium nitrite (NaNO_2_), aluminum chloride (AlCl_3_), chloramphenicol, 2,2-azobis (2-amidinopropane) dihydrochloride (AAPH), fluorescein sodium salt, and 2′-7′dichlorofluorescein diacetate (DCFH-DA) were purchased from Sigma-Aldrich (Spruce St, Saint Louis, MO, USA). Ethanol, methanol, acetone, and acetic acid were purchased from VWR (Radnor, PA, USA), while hydrochloric acid was purchased from Merck (Readington, NJ, USA). 

### 2.2. Bee Pollen Sampling and Palynological Analysis

The organic bee pollen, provided by the Tuscan farm “Sapori Mediterranei”, was harvested using beehives equipped with bottom-fitted pollen traps located in Bagni di Lucca (Latitude 44,0146-Longitude 10,6453) in Lucca Province (Tuscany, Italy). The sample was stored at room temperature until further analysis. The palynological analysis was carried out by Studio Naturalistico Il Pianeta Naturale (Valfabbrica, PG, Italy). The examination under the microscope was carried out at 1800× magnification, and the relative frequencies of each pollen type were determined by counting at least 1000 pollen grains, following five parallel equidistant lines uniformly distributed over the entire observable field [15]. 

### 2.3. Bee Pollen Extraction

Bee pollen extract (50 mg/mL) was obtained by powdering bee pollen grains finely and adding ethanol 95%. The sample was then homogenized using an Ultraturrax (Kinematica Polytron PT MR 2100), incubated for 1 h at room temperature, while being slightly shaken, and centrifuged (Jouan CR31, Newport Pagnell, UK) for 10 min at 2700× *g* at 4 °C. The supernatant was collected and kept at −20 °C in the dark until use. The ethanolic bee pollen extract was used to determine total polyphenols, flavonoids, flavonols, monomeric anthocyanins, and in vitro and ex vivo antioxidant activities. On the other hand, an acetone 80% bee pollen extract (100 mg/mL) was prepared to assess total carotenoids, and a water bee pollen extract (200 mg/mL) was used to evaluate the Fe^2+^ chelation ability. The latter extracts were centrifuged for 10 min at 6620× *g* and the supernatants were collected and kept at −20 °C in the dark until use. 

### 2.4. Phytochemical Characterization

Total polyphenols, flavonoids, flavonols, and monomeric anthocyanins were determined as described by Gabriele et al. [3]. Total polyphenol content, estimated as Folin–Ciocalteu (FC) reducing capacity, was expressed as mg gallic acid equivalent (GAE)/g fresh weight (FW) and the absorbance was read at 760 nm (Perkin Elmer UV/VIS Lambda 365, Waltham, MA, USA). Flavonoids, quantified using the aluminum chloride colorimetric method, were expressed as mg quercetin equivalent (QE)/g FW and the absorbance was recorded at 430 nm. The flavonols content was expressed as mg quercetin equivalent (QE)/g FW and the absorbance was measured at 360 nm following 30 min incubation of 25 µL of ethanolic bee pollen extract with 225 µL of 10% ethanol, 250 µL of 0.1% HCl in 95% ethanol, and 1000 µL of 2% HCl. Total monomeric anthocyanins, assessed using the pH differential method, were expressed as mg cyanidin-3-glucoside equivalents (C3GE)/g FW, using the molar extinction coefficient of 26,900 L cm^−1^ mol^−1^ and molecular weight of 449.2 g mol^−1^, and the absorbance was read at 520 nm and 700 nm. Carotenoid content was determined according to the spectrophotometric method described by Kostić et al. [16]. The absorbance was measured at 450 nm and total carotenoids were expressed as µg carotenoids/g FW using the following formula: µg/g = (A·V·10^6^)/(E1cm·100·m), where A is the absorbance of the sample, V is the sample volume, E1cm is the extinction coefficient of the solvent used (2500), and m is the sample mass. 

### 2.5. Microbiological Characterization of Yeast from Pollen

#### 2.5.1. Isolation of Yeast Strains from Bee Pollen

One gram of bee pollen was used for serial dilutions in 0.9% NaCl according to Chiellini et al. [17]. Each dilution (from 10^−1^ to 10^−4^) was plated in triplicate in Yeast Extract-Peptone-Dextrose (YPD) Agar Medium supplemented with 1 mg/mL chloramphenicol according to Di Paola et al. [18]. Ten different colonies were isolated from the plates and streaked in YPD agar plates supplemented with 1 mg/mL chloramphenicol. The procedure was repeated three times in order to obtain pure strains. Strains were preserved at −80 °C and −20 °C in 20% glycerol.

#### 2.5.2. ITS Amplification and Phylogenetic Analysis

Pure strains in YPD agar plates were used to obtain DNA through thermal lysis according to Di Paola et al. [18]; the obtained DNA was used for the amplification of the ITS1-5.8-S-ITS2 regions (ribosomal Internal Transcribed Spacer, ITS), using primers ITS1 (5′-GTTTCCGTAGGTGAACTTGC-3′) and ITS4 (5′-TCCTCCGCTTATTGATATGC-3′), according to the protocol described in Di Paola et al. [18]. The obtained amplicons were purified by ethanol/EDTA/Na-acetate precipitation according to Chiellini et al. [17], and then sent to an external company for Sanger Sequencing (Mycrosynth, Germany). The obtained sequences were then processed as described in Gabriele et al. [19], and the phylogenetic analysis was conducted with the Maximum Likelihood method on a total of 85 sequences (75 high-quality sequences selected from international databases, and 10 sequences belonging to our yeasts isolates).

#### 2.5.3. Antioxidant Activity of Yeasts

The 2,2-diphenyl1-1-picrylhydrazyl (DPPH) radical scavenging ability of seven yeast strains (one representative of each different genus/species, namely Y1, Y2) Y3, Y5, Y6, Y7, and Y9) was determined using the method described by Ciafardini and Zullo [20], with some modifications. Yeasts were grown in YPD broth supplemented with 1 mg/mL chloramphenicol at 26 °C in agitation overnight. After growing, the optical density was adjusted to an optical density of 600 nm (OD_600_) comprised between 1.1 and 1.5. An aliquot (1 mL) of each yeast culture was centrifuged at 6000× *g* for 5 min, washed, and re-suspended with the same volume of sterile 0.9% NaCl. Next, 400 µL of samples were incubated with 500 µL of DPPH (0.2 M in methanol) for 30 min in the dark and centrifuged for 5 min at 6000× *g* to remove the cells. The absorbance (517 nm) was measured in 96-well plates using a FLUOstar Omega Microplate Reader, (BMG LABTECH, Germany), and the antiradical activity (ARA) was calculated as follows: ARA % = [1 − (A_517_ sample/A_517_ blank)] × 100. Data are the mean ± SD of two independent experiments. Trolox was used as the standard. 

### 2.6. In Vitro Antioxidant Activities of Bee Pollen Extract

#### 2.6.1. ABTS^+^ Radical Scavenging Activity

The 2-2′-azino-bis(3-ethylbenzthiazoline-6-sulfonic acid) radical cation (ABTS^·+^) scavenging activity of bee pollen extract was determined according to Lopez-Martinez et al. [21], with slight modifications. Fresh ABTS^·+^ stock solution (80 µL 140 mM potassium persulfate added to 5 mL 7 mM ABTS) was shaken overnight in the dark, then diluted with methanol to obtain an absorbance of 0.7 ± 0.02 at 734 nm (Perkin Elmer UV/VIS Lambda 365, Waltham, MA, USA). Lastly, 190 µL of bee pollen extract were added to 1 mL of ABTS^·+^ diluted solution, and the absorbance was read at 734 nm after 10 min incubation. The percentage ABTS^·+^ reduction was calculated as follows: % ABTS^·+^ reduction = [(A_i_ − A_f_)/A_i_] × 100, where A_i_ is the initial absorbance and A_f_ is the final absorbance. The ABTS^·+^ scavenging activity was expressed as Trolox equivalent antioxidant capacity (TEAC) using a standard curve of Trolox (5–1000 µg/mL). The bee pollen extract concentration corresponding to 50% of ABTS^·+^ radical inhibition (EC_50_ value, mg/mL) was also determined.

#### 2.6.2. DPPH^•^ Antiradical Activity

The DPPH^•^ antiradical activity of the bee pollen extract was determined according to Chiellini et al. [22]. The antiradical activity (ARA) was calculated as the percentage of DPPH^•^ inhibition using the following equation: ARA % = [1 − (A_S_/A_C_)] × 100, where A_S_ is the absorbance of the sample and A_C_ is the absorbance of the DPPH solution. The bee pollen extract concentration corresponding to 50% of DPPH^•^ radical inhibition (EC_50_ value, mg/mL) was also determined.

#### 2.6.3. Oxygen Radical Absorbance Capacity (ORAC) Assay

The oxygen radical absorbance capacity (ORAC) of bee pollen extract was determined as described by Gabriele et al. [3]. AAPH was used as a peroxyl radicals’ generator and fluorescein as the probe. Fluorescein fluorescence decay was read at λ_ex_ 485 nm and λ_em_ 514 nm using a VictorTM X3 Multilabel Plate Reader (MA, USA). Trolox was used as the standard, and results were expressed as ORAC units (µmol TE/g FW). 

#### 2.6.4. Ferric Reducing Antioxidant Power (FRAP) Assay

The ability of bee pollen extract to reduce ferric iron (Fe^3+^) to ferrous iron (Fe^2+^) was evaluated by the FRAP assay according to Colosimo et al. [23] with slight modifications. Briefly, 85 µL of bee pollen extract was added to 2.5 mL fresh FRAP buffer (300 mM acetate buffer pH 3.6, 10 mM TPTZ in 40 mM HCl, and 20 mM FeCl_3_·6H_2_O at a ratio of 10:1:1). After 30 min of incubation at room temperature, the absorbance was measured at 593 nm (Perkin Elmer UV/VIS Lambda 365, Waltham, MA, USA). Results were expressed as Fe^2+^ equivalents (µM), using a standard curve of FeSO_4_·7H_2_O (15.62–2000 μM), and as μmol Fe^2+^/g FW.

#### 2.6.5. Fe^2+^ Chelation Ability

The ability of aqueous bee pollen extract to chelate Fe^2+^ was estimated using the colorimetric method described by Santos et al. [24] with some modifications. Briefly, 250 µL of bee pollen extract were added to 800 µL ultrapure water and 100 µL 0.3 mM FeSO_4_. After 5 min, 150 µL of 0.8 mM ferrozine were added. The absorbance was read at 562 nm (Perkin Elmer UV/VIS Lambda 365, Waltham, MA, USA) after 15 min of incubation. The results were expressed as mg EDTA equivalent (EDTAE)/g FW using a standard curve of EDTA (3.125–75 µg/mL). A decrease in absorbance is linked to an increase in Fe^2+^ chelating capacity.

### 2.7. Ex Vivo Biological Activities

#### 2.7.1. Preparation of Human Erythrocytes

Erythrocytes were collected from healthy blood donors upon informed consent for the use of residual blood for research purposes according to the Italian regulations and, in particular, the regulations of “Fondazione G. Monasterio CNR-Regione Toscana”. Blood was collected in EDTA-treated tubes and centrifuged at 2300× *g* for 20 min at 4 °C to remove the buffy coat and plasma. The erythrocytes were then washed twice with PBS pH 7.4.

#### 2.7.2. Cellular Antioxidant Activity in Red Blood Cells

The cellular antioxidant activity (CAA) of ethanolic bee pollen extract (BE 25 and 50 µg/mL) was assessed ex vivo on oxidized red blood cells according to the CAA-RBC method described by Frassinetti et al. [25]. The 2,2-azobis(2-amidinopropane) dihydrochloride (AAPH), a free radical generator, was used as an oxidative stressor. Quercetin (Que) was used as the standard, and the fluorescence was recorded at λ_ex_ 485 nm and λ_em_ 535 nm (VictorTM X3 Multilabel Plate Reader, MA, USA). Results were expressed as follows: CAA unit = 100 − (∫SA/∫CA) × 100, where ∫SA is the integrated area of the sample curve and ∫CA is the integrated area of the control curve.

#### 2.7.3. Erythrocytes Oxidative Hemolysis

The anti-hemolytic effects of ethanolic bee pollen extracts (BE 25 and 50 µg/mL) were evaluated on oxidized human erythrocytes as described by Frassinetti et al. [25]. The hemolysis of erythrocytes was induced by the thermal decomposition of AAPH in peroxyl radicals and was read at 540 nm (VictorTM X3 Multilabel Plate Reader, MA, USA). Quercetin (Que) was used as the standard. Results were expressed as a percentage of hemolysis with respect to control, referring to AAPH-treated erythrocytes.

### 2.8. Human Colon Cancer Cell (HT-29) Line Treatments and Viability

The human colonic adenocarcinoma cell (HT-29) line (DSMZ, Germany) was grown, as previously reported [26]. All treatments were carried out using DMEM + F12 medium without phenol red and FBS, containing antibiotics. HT-29 cells were 1 h pre-treated with or without ethanolic bee pollen extract (BE 10 and 50 µg/mL). After that, the cells were stimulated for 4 and 24 h with or without 25 ng/mL TNF-α. HT-29 cell viability was evaluated by the MTT assay as previously described [27]. 

### 2.9. Total RNA Extraction and Quantitative Real-Time PCR (qRT-PCR)

Total RNA was isolated from HT-29 cells using the E.Z.N.A.^®^ Total RNA Kit I (OMEGA bio-tek, Norcross, GA, USA) and reverse-transcribed using the iScript™ Advanced cDNA Synthesis Kit (Bio-Rad, Hercules, CA, USA). A quantitative Real-Time PCR was performed using the SsoFast™ EvaGreen^®^ Supermix (Bio-Rad, CA, USA) in a CFX Connect Real-Time PCR Detection System (Bio-Rad, CA, USA). IL-8 (C-X-C motif chemokine ligand 8), COX-2 (prostaglandin-endoperoxide synthase 2), ICAM-1 (intercellular adhesion molecule-1), and β-actin gene primers were previously described by Gabriele et al. [26]. The gene expression was calculated by the 2^−ΔΔCT^ relative quantification method and data are expressed as a fold-change of expression levels compared to the control.

### 2.10. Statistical Analysis

The results were expressed as mean ± standard deviation (SD) of at least three replicates. A statistical analysis was performed using GraphPad Prism, version 5.00 (GraphPad software, San Diego, CA, USA). Data were analyzed by one-way analysis of variance (ANOVA) with Bonferroni’s or Dunnett’s Multiple Comparison test and a *p*-value lower than 0.05 was considered statistically significant.

## 3. Results and Discussion

### 3.1. Palynological Analysis

The bee pollen sample from Bagni di Lucca (LU, Tuscany, Italy) is composed of more botanical species differently distributed in the analyzed sample. The *Castanea sativa* sp. was the most representative (88.8%), followed by *Hedera helix* sp. (4.2%), *Rubus ulmifolius Schott* sp. (1.7%), and others (5.3%). Each pollen load owned a homogeneous and monospecific pollen content. Considering the high prevalence of *Castanea* pollen grains (>45%), we can classify our sample as a monofloral bee pollen type according to what was reported by Alimoglu et al. [10].

### 3.2. Phytochemical Profile

Several natural polyphenols can be found in plant-based foods, contributing to the organoleptic properties of plants/foods, such as color, odor, flavor, astringency, bitterness, and oxidative stability [28,29,30]. Generally, in plants, polyphenols are produced as secondary metabolites involved in the defense against pathogens, parasites, plant predators, and UV light [28]. Moreover, these bioactive compounds can protect against reactive oxygen and nitrogen species, showing good antioxidant and radical scavenging activities, and exhibit therapeutic properties such as cardio-protective, anti-inflammatory, anti-microbial, anti-aging, and anti-tumor activities [22,28,29].

According to the literature, phenolic compounds are one of the molecular classes most representative in bee pollen samples [1,2,9,31]. However, the phytochemical composition of bee pollen samples, in particular the type and quantity of its bioactive compounds, depends on geographical and botanical origin, soil characteristics, climatic conditions, and beekeeper activities [2,13,32,33].

The sample herein analyzed was screened for the total polyphenol, flavonoid, flavonol, monomeric anthocyanin, and carotenoid content, and the results are listed in Table 1.

The bee pollen extract from Bagni di Lucca contained good levels of polyphenols. The present findings agreed with the literature data reporting values ranging from approximately 5 and 213.2 mg GAE/g FW [3,5,10,33,34,35,36,37,38,39]. This range suggests a wide variability, probably linked to the different botanical and geographical origins of all the analyzed samples. In particular, the total polyphenol content of our sample is similar to that of other Tuscan bee pollens, mainly *Castanea* (24.75 ± 0.78 mg GAE/g FW), *Cistus* (21.19 ± 0.24 mg GAE/g FW), and *Viburnum* (99%) (20.15 ± 0.15 mg GAE/g FW) previously analyzed by Gabriele et al. and Barbieri et al. [3,34]. Moreover, our bee pollen showed higher polyphenolic content than some Portuguese and Spanish bee pollen samples. For example, Feás et al. reported polyphenol values between 12.9 and 19.8 mg GAE/g FW in organic bee pollen samples collected in Portugal [40], while Serra Bonvehì et al. [38], on eleven Spanish bee pollen samples classified as monofloral (*Cistus ladaniferus*), obtained values between 0.87 and 1.46 g GAE/100 g, corresponding to less than half of the polyphenols found in our sample. Furthermore, the flavonoid findings are in agreement with some literature data with values in the range of 0.3–79.21 mg QE/g FW [10,31,33,37,39]. In detail, the Turkish bee pollen samples, from different plant species (*Asteraceae, Fabaceae, Campanulaceae, Cistaceae*, and *Rosaceae*), analyzed by Gercek et al. [31], had an average content of flavonoids (79.21 mg QE/g FW) higher than ours (Table 1). The latter, on the other hand, contained greater flavonoids content than Brazilian monofloral and polyfloral bee pollen samples (0.3–17.5 mg QE/g FW), collected in São Paulo, and analyzed by De-Melo et al. [37]. Similarly, our sample had a higher flavonoid content than Turkish bee pollen of different botanical origins whose values ranged between 3.26 and 11.89 mg QE/g FW [10]. Instead, the flavonols are more than three times higher than those detected in *Castanea* (4.77 ± 0.09 mg QE/g FW), *Cistus* (4.93 ± 0.05 mg QE/g FW), and *Rubus* (2.52 ± 0.14 mg QE/g FW) Tuscan bee pollens collected in Massa Macinaia (LU) [3].

Monomeric anthocyanins are part of the pigments responsible for the purple, red, and blue coloration of fruits, vegetables, and flowers, and exert several bioactivities such as anti-inflammatory effects [41]. The anthocyanin content detected in our sample is comparable to that reported by Gabriele et al. [3] for *Cistus* (57.19 ± 5.84 mg C3GE/L) and *Rubus* bee pollen (53.44 ± 2.36 mg C3GE/L), while it is lower than *Castanea* bee pollen (77.37 ± 2.55 mg C3GE/L) analyzed in the same study.

Lastly, carotenoids are plant pigments responsible, together with flavonoids, for the characteristic yellow/orange color of bee pollen [42]. Carotenoids can reduce free radicals and protect against lipid peroxidation [43]. Moreover, these compounds are differently contained at different levels in bee pollen as described by Almeida-Muradian et al. [7]. Indeed, in ten varieties of bee pollens from Brazil, carotenoids were present in trace amounts to 451.5 μg/g FW. In particular, the carotenoid content of *Bananeira* (15.85 μg/g FW) and *Pyririca Branca* bee pollens (12.38 μg/g FW) was similar to that obtained for our sample. According to the literature, the average carotenoid content determined in various bee pollens is placed in a range between 1.38 μg/g FW and 425.32 μg/g FW [7,44]. 

### 3.3. Identification and Characterization of Yeast Community of Bee Pollen

#### 3.3.1. Taxonomic Characterization of Isolated Yeast Strains

According to the NCBI blast database, the ten isolated yeasts were taxonomically related to seven different genera/species (Table 2), namely *Starmerella bombicola* (Y1), *Candida magnoliae* (Y2), *Metschnikowia* sp. (Y3, Y8, and Y10), *Aureobasidium* sp. (Y4 and Y5), *Pichia guilliermondii* (Y6), *Rhodotorula* sp. (Y7), and *Curvibasidium* sp. (Y9).

**Figure 1:**
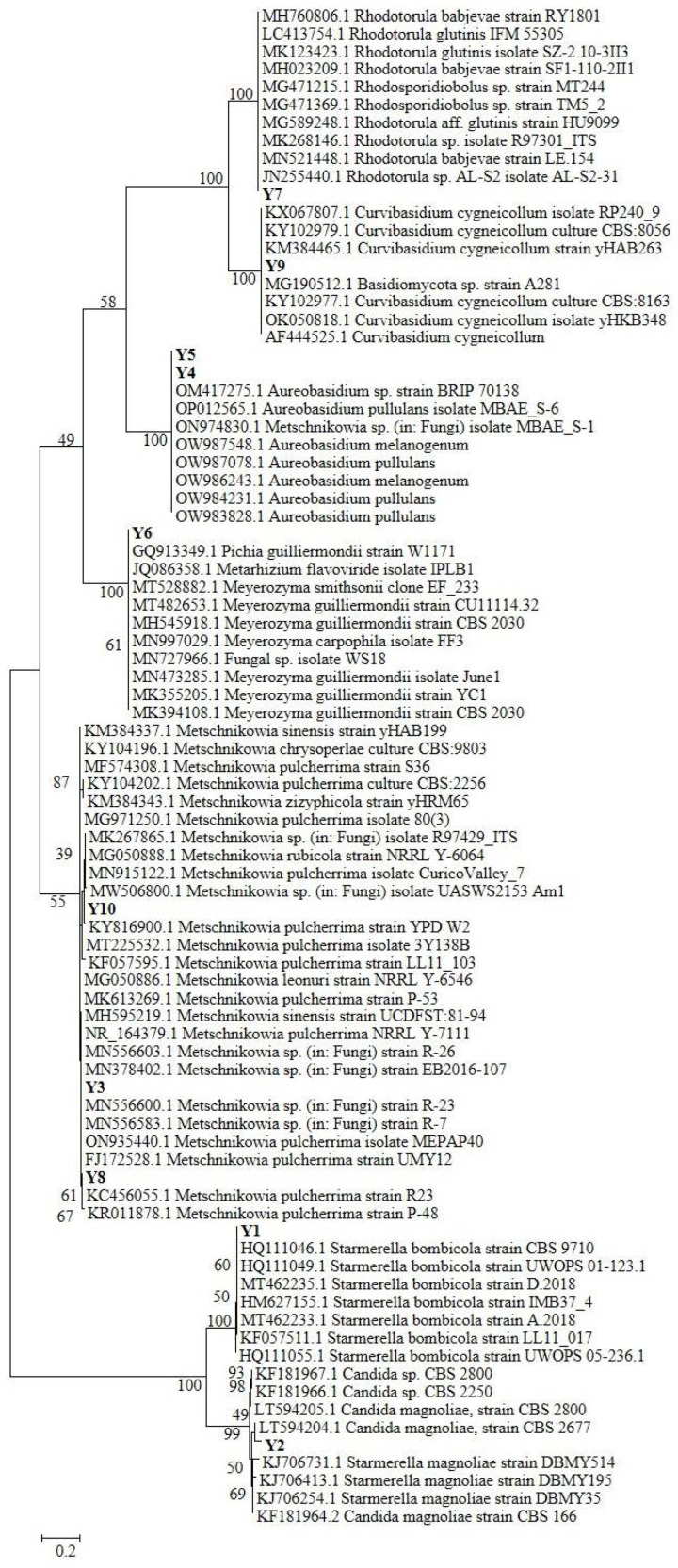
Maximum Likelihood phylogenetic tree reconstruction of the 10 sequences belonging to our yeasts isolates, aligned and compared with 75 high-quality sequences selected from international databases.

The phylogenetic tree reconstruction (Figure 1) allowed a more precise analysis and classification of the isolated yeast strains. While the attribution of Y1, Y2, and Y9 was confirmed for the three species *Starmerella (Candida) bombicola*, *Starmerella (Candida) magnoliae,* and *Curvibasidium cygneicollum* respectively, strains Y3, Y8, and Y10 are all grouped in a clade hosting different *Metschnikowia* sp. species, despite which the most represented is *Metschnikowia pulcherrima*. The same can be assessed for strains Y4 and Y5, both included in an *Aureobasidium* sp. clade with two different species: *A. pullulans* and *A. melanogenum*. According to NCBI Blast, Y6 is closely related to *Pichia guilliermondii (Meyerozyma guillermondii-Candida Guillermondi)*, but the phylogenetic tree attributes this strain to a clade composed of different *Pichia* sp./*Meyerozyma* sp. species, with a strong dominance of species *P. guillermondi*. The same observation can be performed for the Y7 *Rhodotorula* sp.–related strain, which is taxonomically close to both the *R. glutinis* and *R. babjevae* species.

According to the literature, *Rhodotorula* sp. and *Starmerella (Candida) magnoliae* are common inhabitants of bee pollen [45], as well as *Pichia (Candida) guillermondi* [46]. Interestingly, very recent research, evaluating the specialization of yeast during the different phases of bee bread maturation [14], revealed the presence of *Metschnikowia (Candida) rancensis*, *Metschnikowia cf. pulcherrima,* and *Starmerella (Candida) magnoliae* in bee pollen as well, thus again confirming our findings. At the same time, *Starmerella (Candida) bombicola* has been isolated and identified within bee bread in different stages of maturation and from bumblebee honey samples [14]. Furthermore, *Aureobasidium melanogenum* has been retrieved as an important component of bee bread pollen, especially because it showed high potential as a bee probiotic after fermentation with bee pollen [47]. These findings suggest that these strains related to our isolates Y1, Y4, and Y5 have an interaction with the bee pollen production process, and, hence, they might be transferred in our bee pollen sample.

#### 3.3.2. Functional Characterization of Isolated Yeasts

Yeast strains (Y1, Y2, Y3, Y5, Y6, Y7, and Y9) were analyzed from a functional point of view through the DPPH assay. As shown in Table 3, all of them exhibited DPPH^•^ antiradical activity, with the highest DPPH^•^ radical inhibition observed in Y3 (38.02 ± 0.82%), followed by Y9 (32.32 ± 1.21%), and the lowest one was detected in Y1 (22.63 ± 0.95%). Despite the correction with the OD_600_, a similar pattern of inhibition was observed with the exclusion of the Y7 strain (23.06 ± 0.61%), which showed better DPPH^•^ radical scavenging activity. Compared to the antiradical activity of Trolox (6.4–64 µM), showing values ranging from 12.8 to 71.8% DPPH^•^ inhibition, yeast strains showed good antioxidant activity within the interval values of 16 µM Trolox (19.6%) and 32 µM Trolox (44.9%). The DPPH antiradical activity (%) found in this work is in line with those found by Ciafardini and Zullo in yeasts isolated from virgin olive oil; indeed, the authors found values ranging from 28.50 ± 2.12% in strain *Candida diddensiae* 1922 and 83.50 ± 0.71% in strain *Nakazawaea wickerhamii* 1885 [20]. Despite the functional characterization of yeasts highlighting very interesting features and opening new perspectives for their applications in nutraceutical studies, their amount in our bee pollen is low (about 800 CFU/g of bee pollen, data not shown), and is probably not sufficient to conspicuously contribute to the antioxidant characteristics of our sample.

### 3.4. In Vitro Antioxidant Activities

Bee pollen can be considered a functional food due to its rich phytochemical profile and excellent nutraceutical properties, capable of exerting beneficial effects on human health. In fact, bee pollen has been used for years as a food supplement owing to its antioxidant molecules such as carotenoids and phenolic compounds [48]. 

To date, several methods have been developed to evaluate the in vitro antioxidant properties of fruits and vegetables. Indeed, a plant/food extract is a mixture of several compounds having different abilities to terminate radical chain processes for which more than one assay is necessary to determine its antioxidant capacity [49]. In this study, we investigated the in vitro antioxidant capacity and the radical scavenging activity of our bee pollen sample using five chemical assays, including ABTS, DPPH, ORAC, FRAP, and Fe^2+^-metal chelating activity (Table 4), which is expected to cover several mechanisms of action. Among these, the ORAC was used as a hydrogen atom transfer (HAT)-based method, while ABTS, DPPH, and FRAP were used as three mixed-mode electron transfer (ET)- and HAT-based methods [49].

As shown in Table 4, our bee pollen sample exhibited high free radical scavenging capacity with an ABTS^·+^ inhibition value of about 5.31 µM Trolox equivalent corresponding to an EC_50_ value of 0.01 mg/mL of ethanolic extract providing 50% ABTS^·+^ radicals inhibition. As reported in the literature, bee pollen samples usually show higher EC_50_ values, ranging from 0.91–5.73 mg/mL of extract, confirming a good radical scavenging capacity of our sample [33,50,51]. 

On the other hand, the ability of our bee pollen sample to inhibit 50% of DPPH^•^ organic radicals in solution was reported in Table 4 as EC_50_, corresponding to 0.148 ± 0.001 mg/mL of ethanolic extract. In addition, by calculating the ARA %, the bee pollen sample (10 mg/mL) showed inhibition values of 86.8 ± 0.5%. According to Leja et al., the present findings suggest a remarkable anti-radical capacity of our sample [48]. Generally, for bee pollen, the range of EC_50_ values reported in the literature is 0.01–7.99 mg/mL, and the EC_50_ value obtained for our sample, is comparable to or lower than other described pollens, indicating a powerful ability to inhibit DPPH^•^ radicals [3,33,40,50,51,51,52,53,53,54,55,56]. For instance, De-Melo et al., analyzing 56 samples of Brazilian bee pollen of different botanical and geographical origins, obtained EC_50_ values between 0.4 and 7.9 mg/mL of extract higher than that observed for our sample [37]. Similarly, our sample showed a lower value of EC_50_ than that of other Tuscan bee pollens collected in Massa Macinaia (LU), mainly *Castanea* (215.2 μg/mL), *Cistus* (224 μg/mL), and *Rubus* (641.3 μg/mL) [3], and similar to that of Palm bee pollen (0.14 mg/mL) analyzed by LeBlanc et al. [36].

Moreover, ORAC units of our bee pollen are comparable to those of other bee pollens described in the literature with ORAC values in the range of 133–916 μmol TE/g [3,37,57,58]. Instead, the FRAP value corresponding to 300.96 μmol Fe^2+^/g FW, is higher than those obtained by Barbieri et al. [34], ranging from 14.77 to 190.27 µmol Fe^2+^/g, and by Saral et al., who analyzed Turkish bee pollen samples having values ranging from 8.69 and 84.89 μmol Fe^2+^/g [56]. Finally, the Fe^2+^-metal chelating ability of aqueous bee pollen extract was also assessed (Table 4), and the percentage of inhibition of the bee pollen sample (100 mg/mL) of Fe^2+^-ferrozine complex was about 70%. This percentage demonstrates the good ability of our sample to chelate iron, exerting an important antioxidant effect. 

All of these methods give rise to coherent results and, for each one, our bee pollen sample showed good antioxidant activity, free radical scavenging activity, and metal-chelating ability. 

### 3.5. Biological Effects of Bee Pollen on Human Erythrocytes

It is well known that chemical assay results do not always reflect the biological activity of complex compounds and/or extract [59]. Therefore, to study the biological effects of the bee pollen at the cellular level, we evaluated its antioxidant activity on an ex vivo model of human red blood cells exposed to oxidative insult. Erythrocytes have an important role in our body because they protect against antioxidant and anti-inflammatory insults. They have no nucleus and mitochondria and owing to the presence of proteins and polyunsaturated fatty acids on their membrane, which are highly susceptible to oxidation, they represent a suitable ex vivo cellular system to study the radical scavenging capacity of several compounds [2]. Through the CAA-RBC and hemolysis tests, it is possible to induce lipid and protein peroxidation on erythrocytes membranes by the thermal decomposition of AAPH in peroxyl radicals that can damage cells’ membranes and, at high doses, induce cell lysis [2]. 

**Figure 2:**
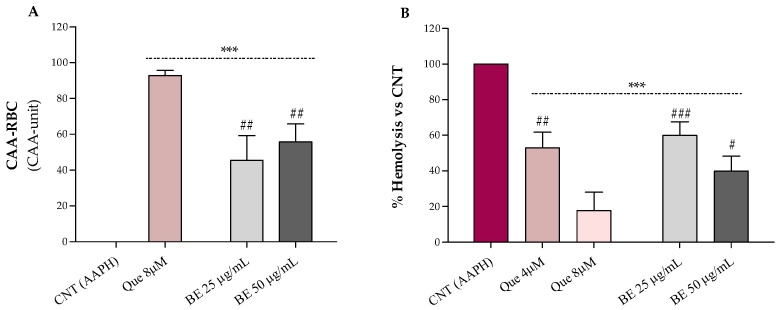
Effect of the bee pollen extract (BE 25 and 50 µg/mL) on the cellular antioxidant activity–CAA (**A**) and AAPH-induced oxidative hemolysis (**B**) on oxidized human erythrocytes. Quercetin (Que) was used as a standard. Results were expressed as mean ± SD. One-way ANOVA with Bonferroni’s Multiple Comparison test: * significantly different from control (CNT, CAA = 0), *** *p* < 0.001; # significantly different from Que 8 µM (corresponding to 2.4 µg/mL): # *p* < 0.05, ## *p* < 0.01, ### *p* < 0.001.

The results relative to the erythrocytes’ antioxidant protection against oxidative stress by bee pollen extract are shown in Figure 2A (CAA-RBC) and Figure 2B (hemolysis test). Bee pollen pre-treatments, at both doses, improved with the antioxidant activity of human erythrocytes by about 55% compared to the control (CAA = 0), with CAA values lower than the quercetin 8 µM (~93%), corresponding to 2.4 µg/mL, which is about 10- to 20-fold lower than the concentrations used for our sample (Figure 2A). These results are higher than what was observed by Gabriele et al. for *Castanea*, *Cistus*, and *Rubus* bee pollen extracts (100 μg/mL), and comparable to those obtained by Barbieri et al. for *Rubus* (90%) and *Eucalyptus* (96%) samples (50 µg/mL) [2,34]. Additionally, in Figure 2B, we can observe that bee pollen treatments exerted a dose-dependent hemolysis inhibition compared to red blood cells exposed to the oxidizing agent alone (AAPH). The bee pollen extract, at both doses, exhibits lower lysis protection than the highest dose of quercetin (8 µM–2.4 µg/mL) but is superimposable to the lowest one (4 µM–1.2 µg/mL), about 20- to 40-fold less concentrated than our sample. In addition, our sample (50 μg/mL) showed comparable % hemolysis inhibition to what was observed by Gabriele et al. for *Castanea* and *Cistus* bee pollen and higher erythrocytes lysis protection than the *Rubus* one [2].

### 3.6. Evaluation of the Anti-Inflammatory Effect of Bee Pollen on Inflamed HT-29 Cells

Inflammation is a complex endogenous process through which our body responds to tissue and cellular damage caused by pathogens, chemicals, and physiological events related to different disorders and pathologies such as tumors, allergies, arthritis, and diabetes [60,61]. Inflammation, induced by these stimuli, consists of a chain of events that causes recognizable symptoms and signs such as swelling, redness, and pain [60]. Furthermore, inflamed tissues release pro-inflammatory mediators, including tumor necrosis factor-alpha (TNF-α) and interleukin 1 (IL-1), which up-regulate and modulate the activity of other cytokines, producing further inflammation that could lead to the onset of other disorders [60]. The anti-inflammatory properties of bee pollen are well-known [1,8,11,43,51,61,62,63]. 

In this study, we evaluated the ability of bee pollen samples from Bagni di Lucca to exert anti-inflammatory effects on the HT-29 cell line exposed for 4 and 24 h to TNF-α, used to induce inflammation. First, to identify the optimal treatment condition and notice possible cytotoxic effects, we performed a toxicity curve using increasing concentrations (1–200 µg/mL) of ethanolic bee pollen extract (BE). The findings demonstrated that HT-29 cell viability was not affected by any doses of bee pollen extract (data not shown).

**Figure 3:**
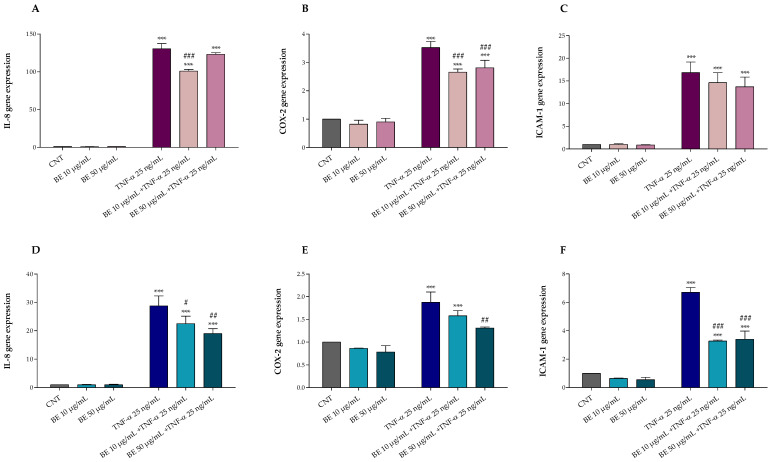
Effects of 1 h pre-treatment with bee pollen extract (BE 10 and 50 µg/mL) on HT-29 exposed 4 h (**A**–**C**) and 24 h (**D**–**F**) to 25 ng/mL TNF-α: (**A**,**D**) IL-8, (**B**,**E**) COX-2, and (**C**,**F**) ICAM-1 (Real-Time qPCR). One-way ANOVA with Dunnett’s Multiple Comparison test: * significantly different from control (CNT), *** *p* < 0.001; ^#^ significantly different from TNF-α: ^#^ *p* < 0.05, ^##^ *p* < 0.01, ^###^ *p* < 0.001.

Furthermore, after 1 h pretreatment with or without BE 10 and 50 µg/mL, HT-29 cells were exposed for 4 and 24 h with or without 25 ng/mL TNF-α. As shown in Figure 3, TNF-α treatment induced inflammation and up-regulated IL-8 (Figure 3A,D), COX-2 (Figure 3B,E), and ICAM-1 (Figure 3C,F) gene expression. Conversely, our results demonstrated that BE pre-treatment was able to protect HT-29 cells against TNF-α induced alterations, mostly at the highest concentration, reducing the IL-8, COX-2, and ICAM-1 up-regulation following 24 h of treatment (Figure 3A–C). 

Comparing the two treatments, after 4 h of exposure to TNF-α, an acute inflammatory response was observed with a gene up-regulation of 2 to 4-fold higher than that observed after 24 h treatment, justifying the lower protective effect of bee pollen at both doses; however, the reduced protection herein observed may also be due to the reduced treatment time which may not be sufficient to activate and provide an adequate cellular anti-inflammatory response.

The anti-inflammatory effect of bee pollen has been investigated sufficiently. Among these, Maruyama et al. demonstrated in vivo the ability of the oral administration of ethanol *Cistus* sp. bee pollen extract to inhibit COX-2 activity and NO production after inducing, through an injection of carrageenan (1%), the formation of edema on the paw of some rats [63]. Moreover, Li et al. studied in vitro the potential anti-inflammatory effect of three lipid extracts of bee pollen (*Brassica campestris* L., *Nelumbo nucifera Gaertn*., and *Camellia sinensis* L.) on RAW 264.7 cells, treated with LPS to induce NO production [64]. In this study it was observed that pre-treatment with bee pollen lipid extracts inhibited the production of NO and significantly reduced the expression of IL-8, IL-10, COX-2, and iNOS, indicating an anti-inflammatory effect, especially with regard to bee pollen lipid extracts having the highest content of phospholipids and unsaturated fatty acids.

Besides modulating the activity of enzymes (e.g., cyclooxygenases, nitric oxide synthase, lipoxygenases) involved in the inflammatory process, polyphenols can decrease the arachidonic acid, prostaglandins, leukotrienes, and nitric oxide production, and regulate the cellular activity of mast cells, lymphocytes, macrophages, and leukocytes [60]. Therefore, these bioactive molecules, in addition to carrying out an antioxidant action and counteracting oxidative stress, can also modulate inflammatory processes [65]. Given that oxidative stress and inflammation are the basis of several chronic diseases and metabolic disorders [65], the bee pollen from Bagni di Lucca could represent a good functional food for human health.

## 4. Conclusions

The high nutritional value and the remarkable phytochemical content make bee pollen one of the most studied bee products. In this work, the bee pollen from Bagni di Lucca (Tuscany, Italy) was characterized from a nutraceutical point of view, evaluating its phytochemical profile, the in vitro and ex vivo antioxidant activities, as well as its anti-inflammatory effects on TNFα-inflamed HT-29 cells. Our results showed good bio-functional activities and a good phytochemical profile of bee pollen, being rich in polyphenols, flavonoids, flavonols, monomeric anthocyanins, and carotenoids. Furthermore, our bee pollen exhibited good in vitro antioxidant activity, good erythrocytes protection from oxidative stress, as well as a fair anti-inflammatory effect. A total of ten yeasts belonging to seven different genera/species were isolated as well: *Starmerella bombicola*, *Candida magnoliae*, *Metschnikowia* sp., *Aureobasidium* sp., *Pichia guilliermondii*, *Rhodotorula* sp., and *Curvibasidium* sp. Interestingly, all of them exhibited DPPH^•^ antiradical activity. In conclusion, the bee pollen from Bagni di Lucca represents an interesting functional food and a potential probiotic product able to exert beneficial effects on human health. 

## Figures and Tables

**Table 1:** Total polyphenol (λ = 760 nm), flavonoid (λ = 430 nm), flavonol (λ = 360 nm), monomeric anthocyanin (λ = 520 and 700 nm), and carotenoid (λ = 450 nm) concentration. Results were expressed as mean values ± SD of three replicates.

	Polyphenols (mg GAE/g FW)	Flavonoids (mg QE/g FW)	Flavonols (mg QE/g FW)	Anthocyanins (mg C3GE/L)	Carotenoids (µg/g FW)
Bee pollen	27.37 ± 1.38	25.37 ± 2.45	13.50 ± 1.57	58.16 ± 1.45	11.78 ± 0.11

**Table 2:** Taxonomic affiliation of sequenced strains according to NCBI Blast analysis. Accession numbers of sequences are provided.

ID Strain	Acc Number	Length (bp)	Identity of the Best BLAST Hit (1° Result)	Identity of the Best BLAST Hit (1° Described Species)
Y1	OP721105	399		*Starmerella bombicola* strain CBS 9710 100% HQ111046.1
Y2	OP723496	360		*Candida magnoliae* strain CBS 2677 96.35% LT594204.1
Y3	OP721106	210	*Metschnikowia* sp. (in: Fungi) strain EB2016-107 99.52% MN378402.1	*Metschnikowia pulcherrima* NRRL Y-7111 100.00% NR_164379.1
Y4	OP721107	384	*Aureobasidium* sp. strain BRIP 70138 100.00% OM417275.1	*Aureobasidium pullulans* isolate MBAE_S-6 100.00% OP012565.1
Y5	OP721108	366	*Aureobasidium* sp. strain BRIP 70138 99.73% OM417275.1	*Aureobasidium pullulans* isolate MBAE_S-6 99.73% OP012565.1
Y6	OP721109	522		*Pichia guilliermondii* strain W1171 98.85% GQ913349.1
Y7	OP721110	499	*Rhodotorula* sp. AL-S2 isolate AL-S2-31 1 99.80% JN255440.1	*Rhodotorula babjevae* strain LE.154 99.60% MN521448.1
Y8	OP721111	228	*Metschnikowia* sp. (in: Fungi) strain R-7 99.56% MN556583.1	*Metschnikowia pulcherrima* culture CBS:5833 99.56% KY104205.1
Y9	OP721112	518	*Basidiomycota* sp. strain A281 100.00% MG190512.1	*Curvibasidium cygneicollum* culture CBS:8163 100.00% KY102977.1
Y10	OP721113	218		*Metschnikowia pulcherrima* strain YPD W2 99.54% KY816900.1

**Table 3:** DPPH^•^ antiradical activity (ARA % standard and corrected for OD_600_) by yeast strains.

ID Strain	OD_600_	ARA (%)	ARA/OD_600_ (%)
Y1	1.40–1.36	22.63 ± 0.95	16.41 ± 1.02
Y2	1.30–1.26	29.08 ± 0.87	22.72 ± 0.18
Y3	1.40–1.40	38.02 ± 0.82	27.16 ± 0.58
Y5	1.30–1.34	29.34 ± 3.35	22.21 ± 2.06
Y6	1.43–1.40	32.11 ± 3.78	22.45 ± 2.64
Y7	1.16–1.11	26.20 ± 1.56	23.06 ± 0.61
Y9	1.51–1.40	32.32 ± 1.21	22.22 ± 0.35

**Table 4:** ABTS, DPPH, ORAC, FRAP, and Fe2+ chelating ability results of bee pollen sample. Results were reported as mean values ± SD of three replicates.

	ABTS (TEAC)	DPPH (EC_50_ mg/mL)	ORAC (µmol TE/g FW)	FRAP (Fe^2+^ µM)	Fe^2+^ Chelation (mg EDTAE/g FW)
Bee pollen	5.31 ± 0.88	0.15 ± 0.00	587.64 ± 21.62	15,054.81 ± 443.19	1.03 ± 0.00

## Data Availability

All of the data is contained within the article.
